# The global financial crisis and health equity: Early experiences from Canada

**DOI:** 10.1186/1744-8603-10-2

**Published:** 2014-01-06

**Authors:** Arne Ruckert, Ronald Labonté

**Affiliations:** 1 Institute of Population Health, University of Ottawa, Room 216A, 1 Stewart Street, Ottawa, ON K1N 6 N5, Canada

**Keywords:** Health equity, Global financial crisis, Social determinants of health, Austerity, Canada

## Abstract

**Background:**

It is widely acknowledged that austerity measures in the wake of the global financial crisis are starting to undermine population health results. Yet, few research studies have focused on the ways in which the financial crisis and the ensuing ‘Great Recession’ have affected health equity, especially through their impact on social determinants of health; neither has much attention been given to the health consequences of the fiscal austerity regime that quickly followed a brief period of counter-cyclical government spending for bank bailouts and economic stimulus. Canada has not remained insulated from these developments, despite its relative success in maneuvering the global financial crisis.

**Methods:**

The study draws on three sources of evidence: A series of semi-structured interviews in Ottawa and Toronto, with key informants selected on the basis of their expertise (n = 12); an analysis of recent (2012) Canadian and Ontario budgetary impacts on social determinants of health; and documentation of trend data on key social health determinants pre- and post the financial crisis.

**Results:**

The findings suggest that health equity is primarily impacted through two main pathways related to the global financial crisis: austerity budgets and associated program cutbacks in areas crucial to addressing the inequitable distribution of social determinants of health, including social assistance, housing, and education; and the qualitative transformation of labor markets, with precarious forms of employment expanding rapidly in the aftermath of the global financial crisis. Preliminary evidence suggests that these tendencies will lead to a further deepening of existing health inequities, unless counter-acted through a change in policy direction.

**Conclusions:**

This article documents some of the effects of financial crisis and severe economic decline on health equity in Canada. However, more research is necessary to study policy choices that could mitigate this effect. Since the policy response to a similar set of economic shocks has globally varied and led to differential health and health equity outcomes, comparative studies are now possible to assess the successes and failures of specific policy responses. This raises the question of what types of public policy can mitigate against the negative health equity effects of severe economic recessions.

## Background

Health equity, broadly defined as the reduction of avoidable and unfair inequalities in health, recently emerged as a central concern amongst a wide range of actors in global health, with the World Health Organization’s (WHO) Commission on Social Determinants of Health (CSDH) playing a pivotal role in its promotion. In its 2008 report, the CSDH concluded that “social injustice is killing people on a grand scale” due to “a toxic combination of poor social policies and programmes and unfair economic arrangements” [1]. This led to a call for meaningful changes to the global economy and global governance arrangements in order to remedy some of the power and wealth imbalances that had accelerated over the last three decades. However, just as the report was released, a severe financial crisis hit the global economy, with a wide range of commentators noting the multiple challenges the crisis is presenting to health [2-5], primarily through its potentially harmful impact on social determinants of health (SDH)^a^.

Few research studies have focused on the ways in which the financial crisis and the ensuing ‘Great Recession’ are affecting health equity and SDH on the ground, notable exceptions being those that have studied European health performance in the wake of the debt crisis [6-10]. Neither has much attention been given to the health consequences of the fiscal austerity regime that quickly followed a brief period of counter-cyclical government spending for bank bailouts and economic stimulus reports [11,12]. None have addressed the situation in Canada, one of the high-income countries frequently cited as having escaped the worst of the financial crisis and its recessionary and austerity effects.

This article builds on a conceptual framework developed to map the various pathways that link the global financial crisis to health equity [13], and expands it to better analyze the impacts of the global financial crisis on health equity in Ontario, the most populous province in Canada. Ontario was strongly impacted by the financial and economic crisis due to its manufacturing sector’s dependence on exports to the United States. The Ontario government recognizes health equity as a policy priority and, notionally, is committed to reducing health inequities through an explicit poverty reduction strategy adopted in 2008 [14], and the introduction of health equity impact (HEIA) assessment tools at the provincial level [15]. Despite these ostensible commitments to reducing health inequities, this article documents how a number of SDH are being negatively affected by the financial crisis’s social and economic impacts and the austerity-driven policy response of the Ontario provincial, and the Canadian federal, governments^b^.

### Outline

**Figure 1:**
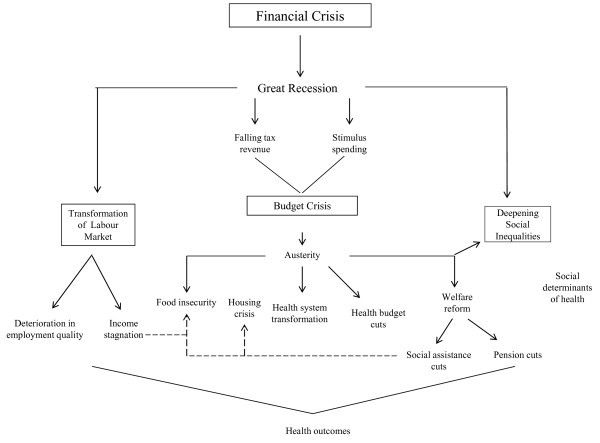
Financial crisis.

The article first introduces the conceptual framework that highlights the pathways that connect the global financial crisis to social determinants of health and associated health equity outcomes (Figure 1). It next focuses on the impact of the global financial crisis on public finances in Ontario, and outlines some of the budgetary and policy changes that have direct implications for health equity, such as cutbacks to education, housing and social assistance programs. The article next focuses on labour market transformations and their potential health consequences. We emphasize that the Great Recession of 2008 had already started to affect health equity negatively through deterioration in SDH, even before most budgetary austerity measures were introduced in 2012. We further argue that different policy choices to Ontario’s austerity agenda are available but remain underutilized due to the unwillingness of the political class to move beyond the currently dominant (neoliberal) economic paradigm. We also note that a different policy response than austerity would be necessary to reverse some of the negative trends observable in the realm of SDH, and that decisions regarding public finance should be at the forefront of critical health research influenced by the SDH paradigm. We conclude with some recommendations about what a social epidemiological research agenda focused on financial and economic crises and their links to population health might look like, and what role public policy interventions might play to mitigate population health risks in times of crisis.

## Methods

The article is based on three main avenues of research: A series of semi-structured interviews in Ottawa and Toronto, with key informants selected on the basis of their expertise on various policy areas related to SDH (n = 12) and comprising active senior government officials, academic researchers and community members with ties to non-governmental organizations (NGOs) and unions; an analysis of recent (2012) Canadian and Ontario budgetary impacts on key SDH that documents macroeconomic and budgetary changes; and documentation of trend data on key SDH pre- and post the financial crisis, including affordable housing, usage of food banks, poverty rate, income trends and employment characteristics. The evolution of SDH as proxy measures for deepening health inequities is justified, given that such inequities generally arise from differential exposures to SDH [16], and that efforts to measure health equity impacts of the financial crisis using standard health indicators such as morbidity and mortality rates would be confounded by lag-time effects. The key informant (KI) portion of the study was approved by the research ethics board of the University of Ottawa, with KIs giving informed consent for use of anonymized quotations.

## Results and discussion

### The global financial crises and health equity: Conceptual clarifications

The global financial crisis is best understood in relation to a number of regulatory and restructuring processes directly linked to the ascendancy of neoliberal policy solutions since the late 1970s, which made global capitalism less resilient and more crisis-prone. With the global integration of finance deepening in the early 1980s, there has been a marked tendency towards financial instability linked to wide-spread capital account liberalization, with more than 200 financial crises transpiring globally over the past three decades [17]. The process of financial deregulation culminated in the global financial crisis of 2008 as the sudden loss of trust in the global banking system evaporated at break-neck speed. This loss of trust was related to the collapse of the US housing market that sent a shockwave through financial markets as it became increasingly clear that many of the financial assets backed by mortgages were worth less than previously assumed [18]. But the deeper underlying root causes of the crisis were the overall inadequate capitalization of banks, lack of transparency in the way in which the financial system operated, for example through so-called over the counter [19] trades that lack any documentation, and relaxation of leverage requirements, allowing banks to hold upwards of 40 times in liabilities what they held in actual assets [20]. While a large body of scholarship on the global financial crisis has emerged, little has been written about how to best connect such a macro-structural phenomenon to health equity outcomes at the local and regional level. Building on a conceptual model that spells out the various pathways that link the global financial crisis to health equity [13], we focus on how the global financial crisis quickly translated into an economic and fiscal crisis, and how this in turn led to the introduction of austerity budgets, with cutbacks in areas crucial to SDH (see Figure 1). We also discuss the indirect impacts of the financial crisis on health through employment-related pathways, especially income stagnation and deterioration in the quality of employment.

Even before the economic impact of the financial crisis started to undermine living conditions, health inequities were widespread in Ontario. Such inequities are well-documented in the areas of life expectancy, infant mortality, and mortality rates related to a number of diseases, as are income-related inequities in incidence and prevalence of various diseases [21]. The existence of wide-spread health inequities is linked, in large part, to the inequitable distribution of SDH, which in turn is influenced by public policy choices. Despite significant analytical capacity in, and a vibrant NGO sector promoting, health equity, Canada lags behind other countries in the implementation of policies that would improve SDH [16,22], instead adopting a number of neoliberal policies creating more inequitable health conditions. Some of the more prominent policy examples implemented over the last two decades include: welfare retrenchment and cutbacks to social assistance programs; regressive tax reforms reducing government revenue and deepening income inequality; labour market reform making employment conditions more flexible and reducing the cost of labour through freezing of the minimum wage; and cutbacks to affordable housing programs and the abolition of rent control. The global financial crisis of 2008 has further deepened these tendencies, especially through the economic and fiscal impact of the crisis, and a jobs recovery that is predominantly driven by precarious forms of employment.

### Economic impact of the global financial crisis on Ontario

As the SDH literature suggests, the health impacts of the Global Financial Crisis (GFC) are mediated by political structures, so that the form of the welfare state mediates the effects of global market forces by determining the extent of state intervention in the economic marketplace [23]. Canada’s liberal welfare regime – in comparison to conservative (e.g. Germany) and social democratic (e.g. Scandinavian countries) welfare regimes – pays less attention to citizen security and welfare provision which translates into lower quality and greater commodification of resources related to SDH. Global events, such as the GFC, thus more directly impact the health of citizens as they are less insulated from market swings and associated social consequences, especially over time. Financial crises are notorious for their long-lasting economic effects. Deleveraging in the financial system can have a dramatic economic impact, currently best evidenced in the European periphery. Canada is largely perceived globally as a safe haven that has been little affected by the financial crisis. Canada’s post-crisis GDP decline has been fairly timid, with a 3.6% drop in economic activity from 2008 to 2009, and the recession lasted only for three quarters [24]. Nevertheless, the budgetary impacts of the crisis have been quite strong and long-lasting, especially in Ontario. Ontario’s budget deficit initially reached more than 6% of GDP in 2009 and by 2012 had declined to around 3% [25]. While the deficit put pressure on the provincial government to respond and return its finances to a more sustainable position, it remains moot whether it is desirable to avoid a deficit at all cost, given the historic low cost of financing government debt (in the case of Ontario at the time of writing at 1.5% on a 3 year fixed-rate bond and 2.8% on a 10 year fixed-rate bond); and especially at a time when there is a glaring ‘social deficit’ in Ontario (discussed in more detail below). There are generally two options to address budget deficits: spending cuts or revenue increases.

From a health equity perspective, the more desirable option to address a budget shortfall would be to raise revenue through progressive forms of taxation and the generation of additional revenue. Accordingly, one informant noted how “we shouldn’t be at all shy about raising revenues, and we probably got a little bit too excited about putting all the action on the expenditure side”, while another identified “a very large over-reliance on spending cuts as opposed to revenue increases” in addressing fiscal shortfalls. However, revenue increases were largely ruled out by the Ontario government which, since the beginning of the crisis, has stated that it will not increase taxes; and this despite corporate and individual taxes, especially for high-income earners and large corporation, having decreased substantively over the past 30 years. For example, corporate tax rates have declined from over 50% in 1970 to 25% in 2012 (combined federal and provincial rates). Similarly, top marginal tax rates in Ontario have decreased from close to 80% in 1970 to 49% by 2013 [26,27]. What is particularly striking is that even since 2000, the ‘own source’ revenues of the province – that is revenues from provincial taxes and fees rather than federal government transfers – have declined from close to 16% to 13.6% of GDP, linked to further cuts in provincial corporate tax rates during this time frame. A government-initiated report to assess the province’s finances recently noted that under an alternative fiscal scenario, with revenue collection returned to the level of 2000, the budget deficit would have been completely wiped out by 2010, with more than CDN $22 billion left over to invest in social programs and services [28]. Yet the only option that was put on the table by the Ontario government to address fiscal shortfalls was to cut back on program spending in a wide variety of areas, including health care spending and program areas directly related to SDH. In consequence, the 2012 budget outlined $17.7 billion worth of program cuts over three years (2012–2015). Facing progressive political opposition, the provincial government ultimately implemented $4 in spending cuts for every $1 dollar in revenue increases, based on a small (2%) increase to the tax rate (surtax) for those in the highest income tax bracket (earning more than $500,000 a year), until the budget is balanced. The overreliance on program cutbacks to address fiscal problems has been described by one informant as “having a larger impact on lower income individuals than on higher income individuals and therefore will widen the gap in health inequities between them”. Another informant noted that “it just seems so incredibly short sighted, especially since we’ve seen the impacts of austerity budgets as they’ve been implemented in the Eurozone. When you actually need to sustain domestic demand you have governments engaging in widespread layoffs and public sector job cuts; that is going to absolutely undermine the economy”.

At the same time, the federal government, under its own austerity drive, is currently limiting the amount of financial transfers to the provinces for health and social programs. This will make it more difficult for provinces to maintain program spending, as they have legislative authority over most social programs but require federal transfers to maintain them. Beginning in 2014, health and social transfers from the federal to the provincial level of government will be maintained at a reduced level. The new formula stipulates that transfers will grow annually by a maximum of 3% (or above, but only if the rate of GDP growth surpasses 3%), compared to the current 6% annual escalator. Provinces that are already generally struggling with larger deficits than the federal government will likely have to make further cuts to health and social services, or limit growth to below inflation (a cut in real terms). In health care alone, total spending as a percentage of GDP in Ontario has fallen in each year since the global financial crisis, with adjusted dollar increases in private spending rising more rapidly than in public spending [29].

### The austerity budget and the SDH scene in Ontario

**Figure 2:**
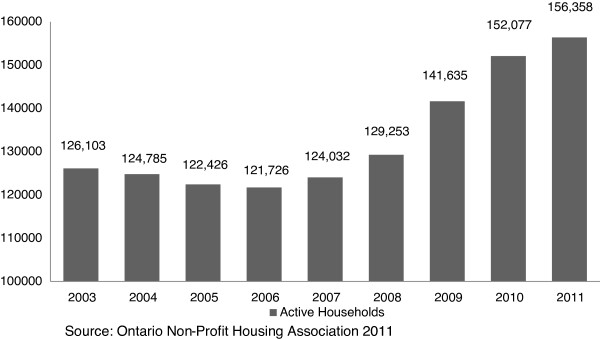
Waiting list for affordable housing in Ontario.

Important SDH pathways other than health care have also been affected by the austerity drive. Housing is widely considered to be an important SDH as housing conditions directly influence an individual’s health through the presence of lead and mold, poor heating and draft, inadequate ventilation, vermin, and other structural conditions [30]; as well as the affordability of housing, given high market rental rates, low social welfare entitlements and the ‘working poverty’ of minimum wage jobs. In 2011, there were more than 150,000 households waiting for affordable (publicly subsidized) housing in Ontario (or 3% of all households in the province), an increase of 17.7% since the beginning of the financial crisis (see Figure 2). Yet, the 2012 budget identifies further cuts to the operating budget of the Ministry of Municipal Affairs and Housing, with spending set at $585.5 million, roughly a 12% reduction from fiscal year 2009 [31]. This means that the operating budget has been cut back in every year since the beginning of the financial crisis in 2008. Operational cutbacks are supplemented by cuts to the capital budget, reducing the capacity for the proper (and healthful) maintenance of subsidized housing units. In addition, cutbacks in the federal budget of 2012 to national housing repair and improvement programs are making a bad situation worse, with an astonishing 97% drop from $674 million in 2011 to $37 million in 2012 [32]. Two other critically important housing programs were also phased out in response to the budget crisis, and will mostly impact those on the margins of society, especially social assistance recipients who lost housing repair and moving subsidies. Reinforcing this observation, one informant suggested that “in the most recent budget (2012), if anything, housing was cut further, so it doesn’t look like there has been any improvement in that area”.

In education, another crucial SDH, the picture is more ambiguous. Although the 2012 federal budget has increased funding for education for Aboriginal communities [32], it has also frozen education transfers to the provinces, leading to a decline over time in inflation-adjusted dollars [33]. Similarly, Ontario will implement a net reduction of educational funding by around $500 million over a three year time frame (2012–2015), including cuts to programs that will likely undermine health equity [32]. For example, Ontario’s cuts to low-impact grants that fund programs such as parenting and family literacy centres will almost certainly have negative health equity implications due to the importance of literacy for healthy behavioural choices. What is more, as one informant noted, financial cutbacks “will lead to a situation where certain families can no longer afford tutoring support, while families who already had their children in private tutoring will remain unaffected”, thus highlighting the inequitable consequences of such cutbacks. Notwithstanding, the Ontario government, despite its general austerity drive, is resolved to phase in all-day kindergarten for 3–5 year olds by 2015, confronting various claims that it cannot afford to do so in the aftermath of the Great Recession and amidst stagnating federal transfers. This represents an important contribution to improving SDH in the province.

Social protection functions as an important financial cushion during times of prolonged unemployment or when the inability to work prevents citizens from meaningfully participating in society and leading healthy lives. While the role of social assistance spending has traditionally been less examined in the SDH literature, a number of recent analyses have found multiple linkages between social assistance spending and population health outcomes [6,34]. The importance of social protection for achieving equitable population health outcomes, especially in times of economic crisis, has also been recently noted in the WHO European Review of SDHs [9]. Given the importance of these programs in supplementing income for the most vulnerable segments of the population, cutbacks in spending will directly undermine health equity goals. In Ontario, the importance of raising social assistance rates was noted by the present government when it developed a province-wide Poverty Reduction Strategy in 2008. After a long period of stagnation, welfare rates in Ontario declined steeply (in constant Canadian dollars), with one informant noting that “people on ODSP and OW^c^ or general welfare, they’re basically receiving approximately 45% less than had the rate continued to go up at a reasonable amount since around 1996. So you’re looking at people’s welfare rates being approximately maybe 55-60% of what they used to be”. Several informants also highlighted that it is impossible to afford a healthy diet and adequate shelter when on social assistance in Ontario, and that people on welfare are far below the official poverty line in Ontario. This is why a provincial Commission for the Review of Social Assistance in Ontario in its 2012 report called for an immediate $100 increase to the social assistance base rate (roughly a 15% increase for singles) [28]. Yet, the 2012 budget limits increases to social assistance rates across the board to 1% (below inflation and therefore a cut in real terms), and limits further increases until the budget has returned to a balanced position. Additional benefits that have a positive impact on the health and quality of life for social assistant recipients have also been trimmed, such as health benefits and special dietary allowances that allow chronically ill people to have a healthy diet.

**Figure 3:**
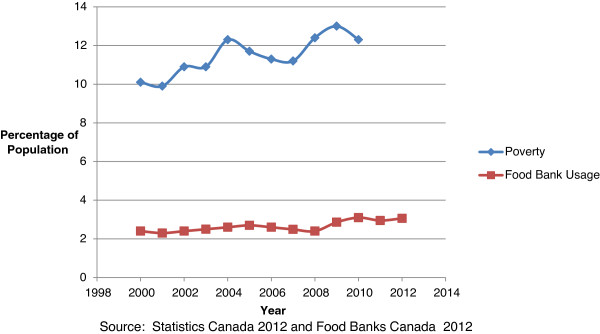
Poverty and food bank usage in Ontario.

Even before the austerity agenda started to take effect in 2012, food insecurity had reached an unprecedented level in Canada. Food security trends in Canada have been measured since 2005 through the Canadian Community Health Survey, with food insecurity defined as lacking access to nutritious food in sufficient quantity and of sufficient quality to maintain good health [35]. Since 2005, food insecurity has been increasing in Canada, with a significant acceleration since the onset of the GFC. By 2011, 12.3% of Canadian households experienced some level of food insecurity, and a rise from 11.3% in 2008, affecting an additional 450,000 Canadians [35]. Growing food insecurity is further manifested through increased usage of food banks. During the month of March 2012, roughly 3% of the population received food from a food bank in Canada, an increase of 2.4% over 2011, and 31% higher than in 2008 [36]. The increase was greatest in Ontario, with roughly 5% of Ontarians (or 650,000 individuals) now considered food insecure [37]. Importantly, working people are increasingly seeking food bank assistance, leading the UN special rapporteur on the right to food to criticize Canada for lacking a proper food security strategy for people living in vulnerable conditions. Poverty rates are also on the rise. Poverty in Canada is generally measured through use of the Low-Income Measure (LIM), a relative measure of poverty that defines the poverty line as 50% of median income after adjusting for family size^d^. The LIM has been trending upwards in Ontario from 8.3% in 1990 to 13.1% in 2010 [38] (see Figure 3).

**Figure 4:**
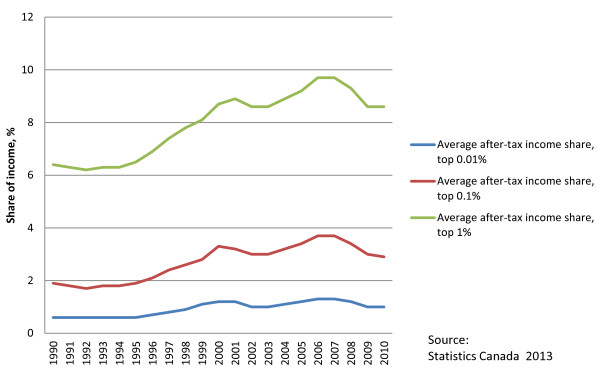
Share of average after-tax income.

**Figure 5:**
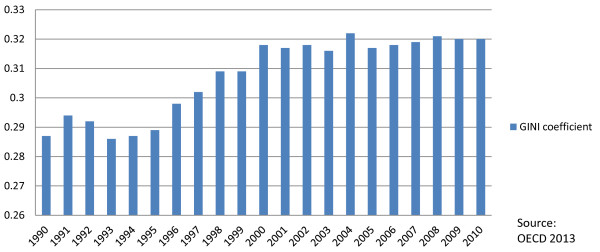
GINI coefficient for after-tax income.

This is happening in a context of growing social inequalities in Canada, including deepening income inequalities as evidenced by a growing share of income going to the top 0.1% and 1% of Canadian income earners (see Figure 4) and a rise in the GINI coefficient over the last two decades (see Figure 5). During this period, income inequality has been growing faster than in all but one other OECD country, with tax policies becoming less effective at reducing inequality [39]. Since the onset of the GFC, the concentration of income in the hands of the richest 1% is down somewhat from the pre-GFC peak; however, the GINI coefficient has decreased only insignificantly. In addition, since 2010, the latest available data on income trends, the stock market has returned to new heights while median family income remains below the 2008 peak (at $57,000 in 2011 compared to $58,100 in 2008), indicating that income inequality has likely deteriorated since then. Due to the fact that the majority of income (around 68%) for people in the lowest income decile is derived from government transfers, the above discussed decline in non-market income support through cutbacks to government transfers will likely further undermine income inequality in the near future. As one informant noted: “If you look at the social determinants of health there’s a range of factors which are currently being undermined…and…the financial crisis is increasing social inequalities and that again will undermine social determinants of health in various areas”.

### The precarization of the Ontario labour market after the financial crisis

That working people are increasingly relying on food banks signals the second major pathway by which the global financial crisis is undermining SDH in Ontario: through its impact on labour markets, especially through the long-standing trend to replace adequate with precarious employment. The Ontario labour market has undergone a dramatic transformation under the pressures of neoliberal globalization, with a steep reduction in manufacturing employment. Employment in this generally unionized and better paid sector fell from over 20% of the workforce in the late 1970s to 12.9% in 2011. Trade (wholesale and retail) and health care are now the two largest sectors of employment in the Ontario economy, with the former commonly associated with low wages and reduced benefits. Many Canadians are resorting to multiple jobs to supplement their low incomes and to increase their experience in hope of landing stable employment in their field. In 2012, 748,200 workers held multiple jobs, compared to 386,300 in 1987 [40]. The rise of different forms of precarious employment in Canada is also obvious through the rise of temporary employment, which serves as a useful, if imperfect, illustration of this on-going labor market transformation. Between 1989 and 2007, temporary employees as a proportion of total employment in Canada rose from 7% to 11% [41]; while the incidence of newly hired employees in temporary positions rose from 11% in 1984 to 21% in 2004 [42]. The number of workers in the greater Toronto area who describe their work as temporary increased by 40% since 1997 [34]. In addition, there are wide-spread inequities in who has access to standard or decent employment, with one informant from the labour movement noting that ”newcomers, for instance, workers of colour, people from marginalized communities, people with disabilities, etc., already had unequal access to what one might call decent jobs or standard work” [before the financial crisis].

The Great Recession has further intensified the trend towards precarious employment in Canada, especially amongst certain population groups. Due to Ontario’s export dependence on the United States, the impact of the financial crisis on the Ontario labour market has been especially strong. Workers who are precariously employed are particularly vulnerable to economic downturns as they are usually the first to bear the consequences of cutbacks and layoffs. But economic recessions also tend to result in compositional changes in job types, intensifying the trend towards greater employment precariousness. Recent research suggests that is happening in Ontario as those generally better insulated from labour market pressures, middle-aged workers with specific skill sets, were not protected from cutbacks in the aftermath of the financial crisis, despite job seniority or implicit lifetime employment relationships [43]. What is more, men bore a disproportionate share of the job losses in sectors that are known to provide permanent full time employment at above average wage rates [43]. In addition, the job recovery in Canada from 2009 to 2012, at closer look, turns out to be less than impressive. Between 2008 and 2011, the majority of job growth in Canada consisted of temporary (222,000) and part-time positions, whereas permanent positions decreased by 50,000 [44]. Even with a strong year of growth in permanent work in 2012, Canada has still not reached pre-recessionary job output, which currently hovers 1% below its 2008 peak at around 62%.

Workers in the low end of the pay and tenure scale – youth, recent immigrants, lone mothers, and workers with little skills, training and education, i.e. those most vulnerable and insecure – have also been affected by the economic downturn. Between 2008 and 2011, the youth employment rate decreased from 60% to 55% and the proportion of recent immigrants working at least 30 hours a week declined from 86.1% to 82.9%, suggesting an increase in precarious employment. The gap between immigrant unemployment levels and those of Canadian born workers has also widened during the Great Recession [45]. Self-employment, which is commonly associated with reduced social protection and income instability, significantly increased in the years following the financial crisis [46]. This has been confirmed by various informants with one arguing that “what’s been happening is that I think the recessions accelerate the general trend of labour market change towards more part-time work, and you see that particularly for youth and seniors, right, the people at the bottom”. This accelerated trend towards precarious employment is concerning because of such work arrangements being unable to serve as a pathway to more secure employment [47]. Further, there is a risk of entrapment, with workers continually cycling through temporary contracts, with little opportunity to move towards greater security. Employment insecurity functions as a chronic stressor and has been identified as an important social determinant of mental health, by triggering arousal of neural and somatic stress responses [48]. The post-financial crisis years have also seen a further erosion in the income potential of precarious workers, evidenced by a steep drop in real average market income of the bottom fifth of all families in Ontario which fell by 23% from 2007 to 2010 [49]. Finally, there is a concern that many of the jobs lost during the recession had (health and other) benefits, while many of the jobs created since the beginning of the financial crisis lack such benefits. For example, one informant noted how “the benefits program for autoworkers has suffered in Ontario, with a two tier system emerging amongst autoworkers”. New hires face increased worker health care co-payments and reduced pensions as car companies are moving from a defined benefit to a defined contribution pension system, where pensions are no longer guaranteed but dependent on market fluctuations.

## Conclusion

Preliminary evidence suggests that the Great Recession and the austerity drive it unleashed are likely to have a significant impact on SDH, with a further deepening in health inequities according to socio-economic status, educational attainment, and geographical location [50]. This means that more research is needed to better document the effects of economic decline on health equity. At the same time, the study of how public policy choices impact the complex nature of SDH and health equity is still in its infancy [51]. However, since the policy response to a similar set of economic shocks has globally varied and led to differential health and health equity outcomes, comparative studies are now possible to assess the successes and failures of specific policy responses. For example, recent evidence from Iceland shows that financial collapse and economic stagnation do not need to translate into adverse health outcomes [7]. This raises the question of what types of public policy can mitigate against the negative health equity effects of severe economic recessions. In the realm of employment, the focus of SDH research should be on how active labour market and social protection policies can better mitigate against employment downturns, and prevent further deterioration of employment quality and standards. It is already apparent that government SDH-related policies, notably the extent of social protection, play a key role in either ameliorating or deepening the impacts of economic recessions. In the case of Canada, our preliminary analysis confirms that the austerity driven policy response will further undermine the equitable distribution of SDH and likely amplify existing health inequities. From the cutbacks to social assistance benefits and declines in housing funding, to the loss of job-related health benefits, the crisis response under way will negatively affect the health of those that can least afford to forego government support.

Alternatives to the current austerity paradigm are readily available. In fact, many economists are warning that the continued application of austerity will make it less likely that Ontario will actually achieve its deficit reduction targets, as austerity starts to undermine the generation of tax revenue by stifling economic growth [33]. One way to avoid falling back into recession would be to focus on much-needed infrastructural investments tackling the ‘social deficit’ in Ontario, by investing in affordable housing, better social protection, and early learning. Given that public debt has never been less expensive, the societal returns on such investments will be rather high, and the population health paybacks of such a strategy would be significant. Finally, ensuring that health equity is at the heart of policy-making will require robust health equity impact assessments of all policies, especially welfare reform. The financial crisis affords us a unique opportunity to reassess and break with the neoliberal project, to select a different path that leads towards a more equitable distribution of SDH. However, this will require better mobilization of the public and better engagement with political elites to move the SDH approach into the mainstream of public health decision-making.

## Endnotes

^a^SDH include the distribution of resources, income, goods, and services, and the everyday circumstances of people’s lives (neighborhood, working conditions, physical environment, education, housing, availability of social assistance, etc.) [1]. The more unequal the distribution of these factors among different population groups remains, the greater the levels of health inequities found in society.

^b^Canada’s division of financing and program authorities/responsibilities between federal and provincial levels means that a policy or taxation change at the federal level often affects the policy space at the provincial level.

^c^The Ontario Disability Support Program (ODSP) is a welfare program that helps people with disabilities who are in financial need to pay for living expenses, like food and housing, The Ontario Works (OW) program is a welfare program that provides temporary assistance in times of financial need and reconnects social assistance recipients with the labor market.

^d^The Gini coefficient for income is another commonly used measure of inequality. One study suggests that reducing this coefficient below 0.3 in OECD countries could reduce adult mortality (15–60 age group) by 9.6% [23]. Canada’s after tax/transfers Gini coefficient was 39.5 in 2010, an historical high; although most of this increase in inequality arose during the 1990s, the country ranked 25th of 30 OECD countries in inequality offsets in the late 2000s. If Canada’s redistributive efforts were to be raised to the OECD average, nearly two thirds of the increase in after-tax inequality since 1981 would be eliminated [47].

## Abbreviations

CSDH: Commission on social determinants of health; GDP: Gross domestic product; KI: Key informant; LIM: Low income measure; SDH: Social determinants of health; WHO: World health organization.

## Competing interests

The authors declare that they have no competing interests.

## Authors’ contributions

RL conceived of the study design and helped to draft the initial manuscript. AR conducted the interviews and SDH trend analysis and drafted the final manuscript. All authors read and approved the final manuscript.
